# Rewarding Volunteering & Enhancing Psychological Meaningfulness in volunteer roles boost Late-Life Well-Being

**DOI:** 10.1093/geroni/igaf122.3496

**Published:** 2025-12-31

**Authors:** Shiyu Lu

**Affiliations:** The University of Hong Kong, Hong Kong, China (People’s Republic)

## Abstract

Although late-life volunteering is considered a health promotion strategy, few studies explored the characteristics of volunteering activities, including the psychological meaningfulness of volunteer roles, the perceived balance between rewards and efforts, and the types of activities that impact the healthy aging process. This study investigates how these dimensions of the volunteering experience influence subjective well-being (SWB) through the lens of belonging and empowerment processes. This cross-sectional study involved 457 volunteers aged 55 and above in Hong Kong from 2023 to 2024. The sense of belonging and general self-efficacy were utilized to capture the belonging and empowerment processes. Results from structural equation models indicated that the perceived balance between efforts and rewards in volunteer roles (β = 0.83, p = 0.002), sense of belonging (β = 0.25, p < 0.001), self-efficacy (β = 0.31, p < 0.001), and engagement in companionship volunteering (β = 1.36, p = 0.005) positively correlated with subjective well-being (SWB) among older adults. The SWB benefits of one-year volunteer hours (β = 0.001, p = 0.032) are mediated by the sense of belonging. Furthermore, the perceived balance between efforts and rewards (β = 0.70, p = 0.001) and the psychological meaningfulness in volunteer roles (β = 1.09, p < 0.001) are jointly mediated by both belonging and empowerment processes. These findings emphasize the significance of achieving a balanced sense of rewards and efforts and psychological meaningfulness in late-life volunteering. This study also highlights specific volunteering activities (e.g., companionship) that can create meaningful volunteering experiences for healthy aging.

